# Incidental Findings on Large-Field-of-View Cone Beam Computed Tomography Scans: Prevalence, Anatomical Distribution and Predictors of Clinical Relevance in a Retrospective Analysis of 203 Patients

**DOI:** 10.3390/diagnostics16193152

**Published:** 2026-09-28

**Authors:** Oliver Heidepriem, Saskia Preissner, Meikel Vesper, Susanne Nahles, Max Heiland, Sameh Attia, Kirstin Vach, Moritz Hertel

**Affiliations:** 1 Department of Oral and Maxillofacial Surgery, Werner Forßmann Krankenhaus, Rudolph-Breitscheid-Str. 100, 16225 Eberswalde, Germany; 2 Department of Oral and Maxillofacial Surgery, Charité—Universitätsmedizin Berlin, Augustenburger Platz 1, 13353 Berlin, Germany; 3 Department of Periodontology, Oral Medicine and Oral Surgery, Charité—Universitätsmedizin Berlin, Aßmannshauser Str. 4-6, 14197 Berlin, Germany; 4 Center for Data Science, Eberswalde University for Sustainable Development, Schicklerstr. 5, 16225 Eberswalde, Germany

**Keywords:** cone-beam computed tomography, incidental findings, retrospective studies, diagnostic imaging, radiography, dental, maxillofacial imaging

## Abstract

**Objectives:** This study aimed to assess the prevalence, anatomical distribution, and clinical relevance of incidental findings in large-field cone beam computed tomography (CBCT) scans and to identify patient-related predictors associated with their occurrence. **Methods:** We conducted a retrospective analysis of 203 patients in a single center who were undergoing CBCT (June 2020–January 2022; field of view: 11 cm × 10 cm) for routine diagnostics or preoperative planning. Findings were categorized by clinical relevance, mapped relative to the primary region of interest (ROI), and analyzed via logistic/Poisson regression for demographic/clinical associations. **Results:** Incidental findings occurred in 79.3% of the patients, with 89.6% outside the ROI. Dental/sinusoidal airway findings were the most common. Increasing age (OR = 1.11; *p* < 0.001) and reduced dentition compared to full dentition (>24 teeth vs full dentition IRR = 1.91; *p* < 0.001 and ≤24 teeth vs full dentition IRR = 2.17; *p* < 0.001) were significant predictors for relevant incidental findings. Other clinical factors showed elevated but non-significant risk estimates. CBCT indication did not predict the occurrence of incidental findings. **Conclusions:** Incidental findings in large-volume CBCT scans are common and frequently located outside the primary ROI. Age and reduced dentition were identified as potential predictors. While these findings may support a more individualized approach to CBCT volume selection, any expansion of the field of view must adhere to established radiation protection principles. Prospective studies are needed to further define risk-adapted imaging strategies.

## 1. Introduction

Cone beam computed tomography (CBCT) has become a fundamental imaging modality in dentomaxillofacial diagnostics due to its ability to provide precise three-dimensional representations of anatomical structures with relatively low radiation doses and moderate costs [1,2]. Nevertheless, given the potential radiation exposure associated with repeated or extensive imaging, there remains a continual focus on dose reduction strategies to minimize patient risk and adhere to the principle of “as low as reasonably achievable” (ALARA)/“as low as diagnostically acceptable” (ALADA) [3,4,5].

One of the most effective approaches to reduce unnecessary radiation exposure is the targeted adjustment of the field of view (FoV), i.e., the scanned field, to the clinically relevant region of interest (ROI). This not only limits the irradiated area but also allows for individual adaptation of the radiation dose to the diagnostic question without substantially compromising image quality or the detection of important incidental findings [6,7].

Although FoV limitation is essential for radiation protection, a restricted examination volume may also limit the detection of findings outside the primary region of interest. This is particularly relevant because the probability of detecting incidental findings is influenced by the amount of anatomy included in the scan. Large-FoV examinations cover several anatomical regions and therefore provide a broader diagnostic overview than limited-FoV examinations, but they also increase the number of structures that require evaluation. A large FoV may consequently increase the likelihood of identifying findings outside the primary region of interest. At the same time, these examinations place greater demands on the examiner, who must assess the entire volume systematically rather than focusing exclusively on the initially suspected pathology. The challenge lies in balancing minimal radiation exposure with comprehensive detection of clinically relevant findings, as excessive FoV reduction may lead to overlooking significant pathological changes outside the focus area. Current recommendations therefore emphasize the use of an appropriate FoV, professional competence, and systematic interpretation of the complete CBCT volume [3,4,5,6,7].

A recent systematic review confirmed that incidental findings occur frequently even in pediatric CBCT datasets, with reported prevalence rates ranging from 0.4% to over 80%, depending on the anatomical region and the evaluation criteria used. Despite methodological limitations in existing studies, the data underscore the diagnostic potential of CBCT beyond the primary indication, particularly when scans are systematically reviewed by trained professionals [8]. Another review focused specifically on CBCT imaging of the maxillofacial region demonstrated that incidental findings vary widely in their clinical significance. Among the included studies, high-significance findings—defined as requiring intervention or referral—were present in up to 31% of cases, whereas low-significance or benign findings accounted for over 70% in some samples. The review further emphasized the absence of professional consensus regarding the interpretation and management of these findings, highlighting the urgent need for interdisciplinary guidelines and structured reporting protocols [9]. Similarly, another recent systematic review focusing on CBCT scans in the head and neck region reported high variability in the prevalence of incidental findings, ranging from 24.6% to 94.3%. The most frequently observed incidental findings included benign airway-related anomalies such as mucous retention cysts and chronic sinusitis, as well as soft tissue calcifications like tonsilloliths or calcified stylohyoid ligaments. Clinically threatening findings, such as carotid artery calcifications, were rare but noteworthy. The review emphasized that, while many incidental findings do not require immediate intervention, CBCT scans must be interpreted by appropriately trained specialists to avoid overlooking potentially significant anomalies [10]. Further insights into the diagnostic ambiguity of sinus-related findings come from a systematic review focusing on the frequency of anatomical variants and pathologies within the maxillary sinuses among CBCT-examined patients undergoing implant-related planning. Based on 23 studies including nearly 12,000 patients, the most frequent variations were sinus pneumatization and septa, while pathological findings such as mucosal thickening, sinusitis, and full opacification showed a prevalence of 7.5% to 66%.

However, the review emphasized the lack of international consensus regarding diagnostic thresholds for sinus pathologies, particularly with respect to mucosal thickening and the radiographic definition of maxillary sinusitis. These inconsistencies highlight the need for standardized evaluation protocols and contextual interpretation of incidental findings [11].

Thus, while CBCT is typically performed for a defined diagnostic purpose, incidental findings beyond the primary region of interest are common and may carry significant clinical implications. Accurate identification and evaluation of such findings are essential for comprehensive patient management and treatment planning. Recent studies demonstrate that differentiated control of FoV in CBCT, when appropriately indicated, achieves significant dose reductions without compromising diagnostic accuracy. Precise definition of the examination area and careful patient selection are essential to ensure both radiation protection and clinical safety. The effective dose in CBCT is influenced by several acquisition-related factors, including field-of-view dimensions, tube voltage, tube current, exposure time, voxel size, and image resolution. Additional technologies, such as automatic exposure modulation and iterative image reconstruction, further optimize radiation dose [3].

Previous studies have investigated incidental findings in CBCT examinations, often including larger patient cohorts. However, the available evidence remains heterogeneous because studies differ in their definitions of incidental findings, anatomical classifications, patient populations, imaging indications, and criteria for clinical significance. In addition, relatively few studies have compared the occurrence of incidental findings across different clinical indications or assessed patient-related factors associated with their number or clinical relevance. The present study addresses this gap by applying a standardized assessment of the frequency, anatomical distribution, and clinical relevance of incidental findings in a defined cohort of large-FoV CBCT examinations. In addition, patient- and examination-related factors associated with the occurrence and number of incidental findings were explored, so that the novelty of the present study lies not in the general investigation of incidental findings in CBCT, but in the focused and standardized evaluation of clearly clinically justified large-FoV examinations across different indications, including both anatomical distribution and clinical relevance as well as patient- and examination-related predictors, in accordance with the ALARA/ALADA principles.

These associations could reflect both cumulative disease burden and susceptibility, and may help guide future decisions on the appropriate extent of radiologic field of view.

We hypothesize that selected patient-related parameters, such as increasing age, male sex, and partial edentulism, may be significantly associated with an increased number of clinically relevant incidental findings in large-field CBCT imaging. These associations could reflect both cumulative disease burden and susceptibility and may help guide future decisions on the appropriate extent of radiologic field of view.

## 2. Materials and Methods

### 2.1. Study Design

This retrospective observational study was conducted at the Center for Implantology, Oral and Maxillofacial Surgery, Hohenzollerndamm 28A, 10713 Berlin, Germany, in cooperation with the Charité—Universitätsmedizin Berlin. All radiological data were derived from routine clinical diagnostics.

The single-center study included large-field CBCT scans (FoV = 11 cm × 10 cm) performed between 1 June 2020 and 31 January 2022, along with the corresponding medical records of the respective patients. All imaging was conducted using standardized acquisition protocols as part of routine preoperative or diagnostic procedures. No additional imaging was performed specifically for the purpose of this study.

The study was designed to evaluate the frequency and distribution of incidental findings within and beyond the primary ROI, and to explore potential predictors for clinically relevant findings that might justify the use of a large FoV in CBCT diagnostics. The Strengthening the Reporting of Observational Studies in Epidemiology (STROBE) statement was taken into consideration during the preparation of this retrospective observational study [12].

### 2.2. Data Source and Patient Selection

All CBCT scans included in this study were retrieved from the digital radiology archive of the aforementioned institution. All datasets that met the predefined inclusion criteria were analyzed. Figure 1 shows the predefined dataset comprised CBCT examinations identified with the indication for large-field-of-view imaging. During screening of this dataset, we identified 203 patients with 208 examinations from which five CBCT examinations met the predefined exclusion criteria. Therefore, not all available examinations fulfilling the inclusion criteria were included in the final analysis. Two examinations were incomplete because wheelchair positioning and elevated shoulders prevented complete rotation of the device around the patient’s head. Three further examinations showed substantial motion artifacts caused by patient instability. All five examinations were repeated, and the repeated scans met the predefined quality and eligibility criteria and were therefore finally included in the final study cohort.

Inclusion criteria were:

- Availability of a large-field CBCT acquired between 1 June 2020 and 31 January 2022;
- Acquisition for a clearly documented clinical indication (e.g., wisdom teeth assessment, apical or endodontic diagnosis, periodontal or orthodontic evaluation, or implant or augmentation planning);
- Sufficient image quality for radiographic evaluation.
- The study protocol also defined the following exclusion criteria:
- CBCTs with severe motion or metal artifacts impairing diagnostic interpretability;
- Duplicate imaging of the same region in the same patient (only the first examination was considered);
- Missing demographic metadata (age or sex);
- Edentulous patients.

However, during the dataset screening process, no CBCT scans were identified that met any of the exclusion criteria. Therefore, all available datasets fulfilling the inclusion criteria were included in the final analysis.

Relevant patient information, including age, gender, imaging indication, and region of interest, was extracted from the electronic clinic management system and pseudonymized prior to statistical evaluation.

Data handling complied with the requirements of the General Data Protection Regulation (GDPR, Regulation (EU) 2016/679), the Federal Data Protection Act (Bundesdatenschutzgesetz, BDSG), and the Berlin Data Protection Act (Berliner Datenschutzgesetz, BlnDSG). No individually identifiable information was available to the investigators at any time.

### 2.3. CBCT Acquisition and Technical Parameters

All CBCT examinations were performed using the ORTHOPHOS SL 3D unit (Dentsply Sirona, Bensheim, Germany), a hybrid imaging system capable of both panoramic and cone beam computed tomography (CBCT) acquisition. The device features a 3D imaging mode with selectable fields of view and variable resolution settings.

The large-field protocol was used consistently for all included scans. In each case, the use of a large FoV was clinically justified by the anticipated treatment approach. Comprehensive imaging of adjacent anatomical areas was required for accurate surgical and prosthetic planning, particularly in the context of complex implant or augmentation procedures. Although the CBCT system allows for FoV reduction, the extended field was deemed necessary to meet diagnostic demands.

According to the manufacturer’s specifications, the maximum available FoV of this device is 11 cm × 10 cm, which was applied to ensure comprehensive visualization of the maxillofacial region. The scans were acquired using a voxel size of 0.2 mm, with a tube voltage of 85 kV and a tube current of 7 mA. The average scan duration was 14 s.

All CBCT data were reconstructed and analyzed using the proprietary Sidexis software (version 4.1.3, 4.2 and 4.3 Dentsply Sirona). The images were assessed in multiplanar reconstruction mode (axial, sagittal, and coronal views) using a standardized viewing protocol. All scans were reviewed in a calibrated diagnostic environment on medical-grade monitors.

### 2.4. Evaluation and Categorization of Findings

All CBCT datasets were systematically reviewed by two experienced senior physicians (OH and MH) for the presence of incidental findings beyond the primary diagnostic objective. Reviewer 1 is a senior surgeon at the Department of Oral and Maxillofacial Surgery, with specialization in oral and maxillofacial surgery, oral surgery and plastic and esthetics surgery and holding the relevant qualifications in CBCT scans and radiation protection. The reviewer routinely evaluates CBCT examinations and computed tomography (CT) scans in daily clinical practice. Reviewer 2 is a dentist specialized in oral surgery and also holds the relevant qualifications in CBCT scans and radiation protection. This reviewer likewise routinely evaluates CBCT examinations in daily clinical practice and serves as an examiner for the state examination in dental radiology at the Faculty of Dentistry, Charité—Universitätsmedizin Berlin.

In cases of disagreement between the two reviewers, a consensus decision was made after discussion. In 94% of the cases, agreement was reached between the reviewers, while consensus decisions were made for the remaining 6%. The final categorization was based on mutual agreement. All evaluations followed a standardized protocol to ensure inter-rater consistency. The evaluation process followed a two-step protocol:

Localization assessment: Each incidental finding was categorized based on its anatomical location as either being within or outside the primary region of interest (ROI) that had initially justified the CBCT acquisition.

Clinical relevance: In a second step, the clinical relevance of each incidental finding was determined. This classification was based on a combination of radiological characteristics and correlating clinical documentation from the patient’s record. Findings were deemed clinically relevant if further diagnostic, therapeutic, or follow-up measures were indicated.

Subsequently, incidental findings were grouped into predefined diagnostic categories:

- Dental findings (e.g., apical radiolucencies and resorptions);
- Skeletal findings (e.g., osteosclerotic foci, foreign bodies, and non-tooth related alterations);
- Sinusoidal/airway findings (e.g., retention cysts, mucoceles, chronic sinusitis with mucosal thickening, nasal septum deviations, and turbinate hypertrophy);
- Other pathologies (e.g., carotid artery atherosclerosis and tonsilloliths).

To ensure diagnostic accuracy, incidental findings were only recorded when the relevant anatomical region was completely visualized in the CBCT dataset. In particular, sinus-related findings were only assessed if the maxillary sinuses were included in the FoV.

### 2.5. Assessed Variables

The following parameters were recorded and included in the statistical analysis. For clarity, the variables are grouped into three categories:

- Demographic variables
  ○ Age at the time of imaging (in years);
  ○ Gender (female/male).
- Diagnostic variables
  ○ Indication for CBCT, categorized as follows:
    ▪ Wisdom teeth position;
    ▪ Apical and endodontic diagnosis;
    ▪ Periodontal diagnosis;
    ▪ Orthodontic assessment;
    ▪ Implant or augmentation planning.
  ○ Localization of the primary ROI, assigned to one or more of the four quadrants (Q1–Q4).
- Clinical variables
  ○ Dentition status, categorized as:
    ▪ Full dentition (28 or more teeth, third molars excluded);
    ▪ Partial dentition:
      ▪ 24 teeth (25–27 teeth);
      ▪ ≤24 teeth (24 teeth or fewer).
  ○ Presence and number of root canals treated and/or apicoectomized teeth.
  ○ Alveolar bone loss, including:
    ▪ Presence of bone loss (yes/no);
    ▪ Severity, categorized according to vertical extension relative to the root length as:
      ▪ Coronal third;
      ▪ Middle third;
      ▪ Apical third.

### 2.6. Statistical Analysis

For this exploratory study, all available datasets from a 1.7-year period containing usable findings and imaging material were used.

Descriptive statistics were calculated to summarize demographic and clinical characteristics of the study population. For all analyses, the patient was considered a statistical unit and only data from a single visit per patient were considered. For categorical variables, cross-tabulations were created, and Chi-square and binomial tests were used to assess statistical associations between independent variables and the presence of incidental findings.

To evaluate the influence of various parameters on the occurrence and frequency of incidental findings, logistic and Poisson regression models were applied. Univariate logistic regression was used to estimate the odds of detecting clinically relevant incidental findings based on the following covariates: age, gender, CBCT indication, and dental status (fully dentate, partially dentate, or edentulous). Univariate Poisson regression was performed to model the number of incidental findings per patient, taking into account, in addition to the covariates mentioned above, whether one or more ROIs were examined. In case of multiple testing, Scheffé’s method was applied for correction of *p*-values.

To test whether incidental findings show associations with the demographic data, a logistic model containing age and sex was applied. Furthermore, for analyses of diagnostic indication for CBCT acquisition and periodontal bone loss, an adjustment for age was done.

All statistical analyses were performed using Stata (version 17.0, StataCorp, College Station, TX, USA). Statistical significance was defined as *p* < 0.05.

Artificial intelligence tools were used solely to support language editing, proofreading, and manuscript formatting. The authors remain fully responsible for the accuracy, interpretation, and final content of the manuscript. No artificial intelligence tool was used for data collection, statistical analysis, image interpretation, or the generation of scientific conclusions.

### 2.7. Ethical Considerations

The study was approved by the Ethics Committee of Charité—Universitätsmedizin Berlin (approval number: EA1/036/26). All procedures were conducted in accordance with the ethical standards of the institutional and national research committees and with the principles of the Declaration of Helsinki (2013 revision).

This investigation was based exclusively on pseudonymized retrospective data. All CBCT images and clinical datasets had been acquired for diagnostic purposes as part of routine clinical care, and no additional imaging or intervention was performed for this study.

## 3. Results

### 3.1. Study Population

The study cohort consisted of 203 patients who underwent CBCT between 1 June 2020 and 31 January 2022 for various dental indications, aged between 18 and 79 years (median age of 37 years). The distribution of subjects included 80 males and 123 females. The indications for CBCT were categorized as follows: wisdom teeth assessment (*n* = 96), apical and endodontic diagnosis (*n* = 24), periodontal diagnosis (*n* = 18), orthodontic assessment (*n* = 7), and implant or augmentation planning (*n* = 58). The mean age varied across indication groups, with wisdom teeth cases being younger on average compared to implant planning. The study cohort comprised 203 patients after exclusion of incomplete data. Demographic and baseline clinical characteristics are summarized in Table 1.

### 3.2. Categorization and Prevalence of Incidental Findings

Incidental findings were categorized into dental, skeletal, sinusoidal/airway, and others. Distribution and frequency are shown in Table 2. Overall, dental findings were the most frequent category (52.2%). Sixty-six percent of the findings were identified as relevant and required treatment, follow-up, or further diagnostic evaluation to ensure appropriate patient care.

### 3.3. Incidental Findings Outside the Region of Interest

The majority of incidental findings were located outside the ROI. Within the group of findings, sinusoidal/airway findings had the highest percentage, with 93.2% of incidental findings located outside the ROI (Table 3).

### 3.4. Prevalence of Incidental Findings by Indication

The frequency of incidental findings varied by CBCT indication, with implant planning cases showing the highest percentage for incidental findings (94.8%), followed by apical and periodontal diagnosis (83.3%). Wisdom teeth (69.8%) and orthodontics (57.1%) show the lower percentages of incidental findings (Table 4).

### 3.5. Prevalence of Incidental Findings Outside the ROI by Indication

The prevalence of incidental findings located outside the defined ROI varied according to the clinical indication for CBCT examination (Table 5; Figure 2). Implant planning cases exhibited the highest percentage of incidental findings outside the ROI (91.5%), followed by apical diagnosis (83.3%) and periodontal diagnosis (66.6%). Wisdom teeth and orthodontic indications showed somewhat lower frequencies.

Figure 2 provides a visual summary of these differences, highlighting the distribution of incidental findings inside versus outside the ROI for each CBCT indication group. For wisdom teeth, implant planning, and apical diagnosis, the proportion of incidental findings located outside the primary ROI was significantly higher than the proportion detected within the ROI. As illustrated, the majority of both intra- and extra-ROI findings were classified as clinically relevant.

Figure 3 shows that the number of incidental findings does not increase with the number of ROIs examined, as one might expect.

### 3.6. Distribution of Incidental Findings Inside vs. Outside the ROI by Indication

A binomial test demonstrated that, within the indication groups of wisdom teeth assessment, implant planning, apical diagnosis, and periodontal evaluation, the proportion of incidental findings located outside the primary ROI was significantly higher than the proportion detected within the ROI (*p* < 0.05). The results are displayed in Table 6.

### 3.7. Association of Demographic Variables with Relevant Incidental Findings

A logistic regression model with the covariates age and gender identified age as a significant predictor of incidental findings (OR = 1.11; 95% CI: 1.06–1.17; *p* < 0.001), while gender showed no difference (OR = 0.93; 95% CI: 0.37–2.30; *p* = 0.874; Table 7). Figure 4 illustrates how the distribution of incidental findings varies with patient age and further differentiates between relevant and non-relevant findings within different CBCT indications. A Poisson regression analyzing the number of incidental findings confirmed significant differences between CBCT indications (*p* = 0.004) and a higher number of findings with increasing age (*p* < 0.001). These results remained consistent when the analysis was restricted to clinically relevant incidental findings located outside the primary ROI.

In the univariate negative-binomial analysis, increasing age was associated with a higher number of incidental findings (IRR = 1.03, 95% CI 1.02–1.03, *p* < 0.001). The number of findings differed significantly between CBCT indications (global *p* < 0.001). Scheffé-adjusted pairwise comparisons showed higher counts for implant planning than for third-molar assessment (IRR = 2.81, 95% CI 1.98–3.97, *p* < 0.001), apical assessment than for third-molar assessment (IRR = 1.98, 95% CI 1.23–3.20, *p* = 0.001), and bone-related indications than for third-molar assessment (IRR = 1.963, 95% CI 1.15–3.34, *p* = 0.004). The results of the negative-binomial sensitivity analysis are summarized in Table 8. They were comparable to those of the Poisson model.

### 3.8. Association of Diagnostic and Clinical Variables with Relevant Incidental Findings

Significant associations were observed between certain diagnostic and clinical variables and the presence of incidental findings located outside the primary region of interest (ROI). Logistic regression analysis adjusted for age revealed that the diagnostic indication for CBCT acquisition was not significantly associated with whether the findings outside the region of interest were relevant or non-relevant (*p* = 0.762). Univariate analyses showed that patients with reduced dentition (>24 teeth and ≤24 teeth) had a significantly higher incidence rate ratio (IRR) of incidental findings compared to fully dentate patients (>24 teeth IRR = 2.40; 95% CI: 1.79–3.22 vs ≤24 teeth IRR = 3.32; 95% CI: 2.48–4.44). In both groups with reduced dentition compared to fully dentate individuals, high statistical relevance was found (*p* < 0.001). Due to the small number of patients with a history of apicoectomy (*n* = 20), no reliable estimation of the association with incidental findings was possible. Patients with the presence of root canal-treated teeth showed an elevated univariate estimated OR of relevant incidental findings (OR = 9.24; 95% CI: 3.02–28.27). Given the limited subgroup sizes, no stratification by the number of root canal-treated teeth was performed. Periodontal bone loss (adjusted for age) compared to non-periodontal bone loss showed an increased OR (OR = 2.17; 95% CI: 0.45–10.54) but this association did not reach statistical significance (*p* = 0.336). The association of clinical variables with incidental findings is shown in Table 9.

## 4. Discussion

This study aimed to evaluate the prevalence, anatomical distribution, and clinical relevance of incidental findings in large-field CBCT scans, and to identify patient-related factors associated with an increased likelihood of such findings. Based on the hypothesis that variables such as age, sex, and dental status may significantly influence the occurrence of clinically relevant incidental findings, the analysis sought to explore potential predictors that could inform future decisions regarding the appropriate selection of field of view in CBCT imaging.

### 4.1. Findings in Context: Relevance and Consistency with Previous Research

The observed prevalence of incidental findings in our study aligns well with the general trend reported in the literature, both in terms of absolute frequency and anatomical distribution. Comparable methodologies and sample sizes further underscore the consistency of our findings with prior studies. However, it must be noted that the reported prevalence rates of incidental findings in CBCT imaging vary widely across the literature, ranging from less than 1% to over 90%. This extraordinary variability reflects the considerable methodological heterogeneity among published studies, including differences in study design, imaging protocols, population characteristics, classification criteria, and definitions of clinical relevance. Such heterogeneity limits the direct comparability of results and complicates the derivation of generalized conclusions [8,10,11,13,14,15,16,17,18,19,20,21]. Additionally, nearly all available studies—our own included—are retrospective in nature, which imposes inherent constraints on the interpretation of findings and precludes causal inference. Despite these limitations, our data support the growing body of evidence suggesting that incidental findings are common, especially in large-field CBCT scans, and may carry clinically relevant implications, particularly when systematically assessed by trained professionals.

Although previous studies have often included larger CBCT cohorts, direct comparison with the present study is limited by substantial methodological differences. Previous investigations varied with respect to patient selection, imaging indications, anatomical coverage, FoV dimensions, definitions of incidental findings, and criteria for clinical relevance. The present study therefore complements rather than replaces these larger prevalence studies by focusing on a clinically defined cohort of large-FoV examinations performed in a specialized surgical setting. Its specific contribution lies in the standardized assessment of the anatomical distribution and clinical relevance of incidental findings across different clinical indications, together with an exploratory analysis of patient- and examination-related factors [9,10,15,16].

In our cohort, incidental findings were detected in over 80% of patients, with dental and sinusoidal/airway-related anomalies being the most frequently observed categories. Notably, nearly 90% of all incidental findings—and an equivalent proportion of those deemed clinically relevant—were located outside the original diagnostic ROI. This result aligns with prior research indicating that a substantial proportion of diagnostically meaningful incidental findings may remain undetected when the selected field of view is too narrow. Moreover, logistic regression analysis and Scheffé’s method identified increasing age and reduced dentition compared to fully dentate patients as significant predictors of relevant findings, which could indicate a higher cumulative disease burden in these subgroups. In the univariate analysis, age was significantly associated with the number of incidental findings. The number of findings also differed significantly between the CBCT indications, with the most pronounced differences observed between implant-planning and third-molar examinations. The sensitivity analysis using a negative-binomial model yielded results that were comparable to those of the Poisson regression.

Other examined clinical variables, such as a history of root canal treatment, showed inconclusive association and failed to reach statistical significance, likely due to limited subgroup sizes and should therefore be interpreted cautiously.

Contrary to our a priori hypothesis, the male sex was not associated with clinically relevant incidental findings in the present cohort. The confidence interval includes both a potentially relevant decrease and an increase in odds, indicating considerable uncertainty around the estimated effect. This finding suggests that sex alone may not be a meaningful predictor of clinical relevance in large-field-of-view CBCT examinations.

Taken together, these findings provide further evidence that patient-specific risk factors may help identify individuals with an increased likelihood of harboring clinically relevant incidental findings—potentially informing future imaging strategies. However, the high proportion of extra-ROI findings also raises important questions regarding the diagnostic sufficiency of reduced FoV protocols in selected clinical scenarios and underscores the need for a nuanced balance between diagnostic comprehensiveness and radiation safety—with radiation protection taking precedence whenever a smaller field of view is sufficient to answer the clinical question.

### 4.2. Methodological Considerations and Limitations

Several limitations inherent to the retrospective design of this study must be acknowledged. First, the analysis was based exclusively on pre-existing clinical and radiological data obtained during routine care. Consequently, the completeness and consistency of documentation—particularly in relation to clinical findings and follow-up decisions—may have varied across cases. This limits the ability to comprehensively verify the diagnostic or therapeutic implications of certain incidental findings.

Second, the classification of incidental findings as clinically relevant was based on a combined assessment of radiographic features and corresponding clinical documentation. Although this approach aimed to reflect real-world decision-making, it inevitably involves a degree of interpretative subjectivity. In the absence of standardized prospective follow-up, there remains a potential risk of misclassifying the true clinical significance of certain findings—particularly those whose relevance depends on individual patient history or subjective interpretation, such as mucosal thickening or minor skeletal anomalies.

Third, although the two reviewers showed a raw agreement of 94% in the classification of clinical relevance, no chance-corrected inter-rater reliability statistic, such as Cohen’s kappa, could be calculated because the individual rating data and the underlying agreement table were not retained. Given that this classification formed the basis for the subsequent analyses, the absence of a formal reliability statistic limits the assessment of the robustness of the outcome classification.

However, all evaluations were carried out by experienced clinicians following a standardized review protocol, and in cases of initial disagreement, findings were jointly discussed until a consensus was reached. Moreover, it was not possible to retrospectively assess whether the clinically relevant incidental findings identified in the extended FoV would have also been detected through alternative diagnostic approaches, such as clinical examination or conventional two-dimensional radiography performed before or after the CBCT scan. As a result, the actual diagnostic benefit of using a large field of view in these cases remains uncertain.

Fourth, the sample size was determined by the availability of eligible archival examinations rather than by an a priori power calculation. Although nearly all available eligible cases during the predefined study period were included, the final sample of 203 examinations may have provided limited statistical power to detect small or moderate associations. This limitation is particularly relevant because several patient- and examination-related variables were evaluated, including age, sex, dental status, clinical indication, and the clinical relevance of incidental findings. Accordingly, non-significant results should not be interpreted as evidence that no association exists, because insufficient power may have contributed to the absence of statistical significance. Conversely, statistically significant findings should be interpreted cautiously because the analyses were exploratory and estimates may be unstable in smaller subgroups. The findings should therefore be regarded as hypothesis-generating and require confirmation in larger, preferably multicenter studies with prospectively defined sample size calculations and prespecified outcomes.

Fifth is the potential for confounding by indication. The assessment of clinical relevance was partly based on the clinical records that also documented the indication for CBCT imaging, and the reviewers were not blinded to this indication. Consequently, variables such as age and dentition status may have been associated with both the reason for referral and the probability of detecting or classifying an incidental finding as clinically relevant. For example, patients referred for implant planning may systematically differ from those referred for third-molar assessment. Therefore, the associations observed in the predictor analysis may partly reflect referral patterns and indication-related differences rather than generalizable biological relationships. These findings should consequently be interpreted as exploratory and hypothesis-generating. Future prospective, multicenter studies with standardized definitions of clinical relevance and blinded assessment independent of the referral indication are needed to address this potential bias.

Finally, a potential source of uncertainty concerns the definition and quantification of the primary region of interest (ROI). Although the examinations were assigned to predefined clinical indication groups, the corresponding primary ROIs were heterogeneous and were not defined as standardized geometric or three-dimensional volumes. Depending on the indication, the target region could comprise an individual tooth, adjacent alveolar bone, a dental quadrant, a cystic or inflammatory lesion, or several contiguous anatomical structures. For example, in cases referred for the assessment of a suspected apical lesion, the indication may be related to a specific tooth or associated symptoms, whereas the actual three-dimensional extent of the lesion and its relationship to adjacent structures can only be established through imaging. Thus, the clinical indication defined the general diagnostic region but not necessarily a precisely measurable three-dimensional target volume.

Because the study was retrospective, the indication-specific ROIs had not been prospectively documented using standardized three-dimensional boundaries. Retrospective assignment of exact ROI limits would therefore have required assumptions and could have introduced additional observer-dependent variability. Consequently, the geometric relationship between the primary ROI and the standardized 11 cm × 10 cm FoV could not be quantified reliably. We acknowledge this as a methodological limitation. The high proportion of findings classified as being outside the primary ROI should therefore be interpreted as describing the anatomical distribution of findings within the acquired volume, rather than as a direct measure of the geometric excess or diagnostic benefit of the large FoV.

### 4.3. Outlook and Future Directions

The high frequency of clinically relevant incidental findings observed in the existing literature [8,10,11,13,14,15,16,17,18,19,20,21] and reflected in our dataset underscores the potential diagnostic value of large-field CBCT imaging, even beyond the primary clinical indication. However, this stands in direct contrast to the principles of radiation protection and highlights the inherent tension between comprehensive diagnostics and adherence to the ALARA/ALADA principles. Further prospective studies with larger cohorts and standardized reporting protocols are needed to define clinical thresholds and risk profiles that may justify the use of extended fields of view in selected cases.

In the long term, the establishment of evidence-based guidelines for CBCT field selection—potentially stratified by indication, age, dental status, and other patient-specific risk factors—would contribute to more consistent and safer diagnostic strategies in dentomaxillofacial imaging. Additionally, emerging technologies such as artificial intelligence and deep-learning algorithms may support the automated detection and triage of incidental findings in the near future, enabling both improved diagnostic sensitivity and more efficient use of radiological resources [22,23,24].

### 4.4. Clinical and Ethical Implications

While the present data suggest that large-field CBCT scans may detect a considerable number of clinically relevant incidental findings, this should not be misconstrued as a general endorsement of routinely using an extended field of view. Any consideration to expand the -FoV beyond the primary diagnostic region must be based on clearly defined clinical scenarios and risk profiles and should always comply with the fundamental principles of radiation protection, including ALARA and ALADA.

Importantly, our study design does not allow for determining whether the identified incidental findings would have been detected by other diagnostic means, such as clinical examination or conventional radiography. As such, the true diagnostic added value of a larger FoV remains subject to further prospective evaluation and must not be overestimated based on retrospective associations alone.

Particularly in pediatric and adolescent patients, ethical considerations become even more critical. According to current recommendations [25], the use of CBCT in younger populations must be strictly limited to the smallest diagnostically justifiable field, ideally in combination with ultra-low-dose protocols and protective measures such as thyroid shielding. The same guidelines clearly emphasize that incidental findings of clinical significance are rare in children and adolescents, and therefore, an expanded FoV cannot be justified as a screening measure in this population.

In light of these considerations, it is evident that further research and interdisciplinary dialog are needed to establish robust clinical guidelines for the appropriate use of CBCT field sizes. Moreover, advances in artificial intelligence and automated image analysis may soon provide valuable tools to support standardized detection and classification of incidental findings—potentially improving diagnostic efficiency without compromising patient safety.

## 5. Conclusions

This retrospective study demonstrated a high overall prevalence of incidental findings in large-field CBCT scans, the majority of which were located outside the primary ROI. Increasing age and reduced dentition were significantly associated with the occurrence of clinically relevant incidental findings, suggesting that cumulative age-related and structural factors may increase the likelihood of unexpected pathology visible on CBCT imaging. Other clinical variables, including prior endodontic or apical surgery, showed no statistically significant associations, likely due to limited subgroup sizes.

These findings highlight that certain patient characteristics—particularly older age and a history of tooth loss—may help identify individuals in whom clinically relevant incidental findings are more likely to occur. While this may justify consideration of a larger field of view in selected high-risk cases, such decisions must remain firmly grounded in the principles of radiation protection and clinical justification. Any potential diagnostic gain must be carefully weighed against the additional radiation exposure and the ethical obligation to minimize patient dose.

The observed associations between age, dentition status, and incidental findings should be interpreted cautiously, as they may partly reflect referral patterns and differences between clinical indications rather than an indication-independent risk profile.

Ultimately, prospective clinical studies using standardized and indication-independent outcome assessment are required to validate these associations and to define evidence-based thresholds for adapting the CBCT field of view. Only through such data can individualized, indication-specific imaging protocols be developed that reconcile diagnostic comprehensiveness with patient safety.

## Figures and Tables

**Figure 1:**
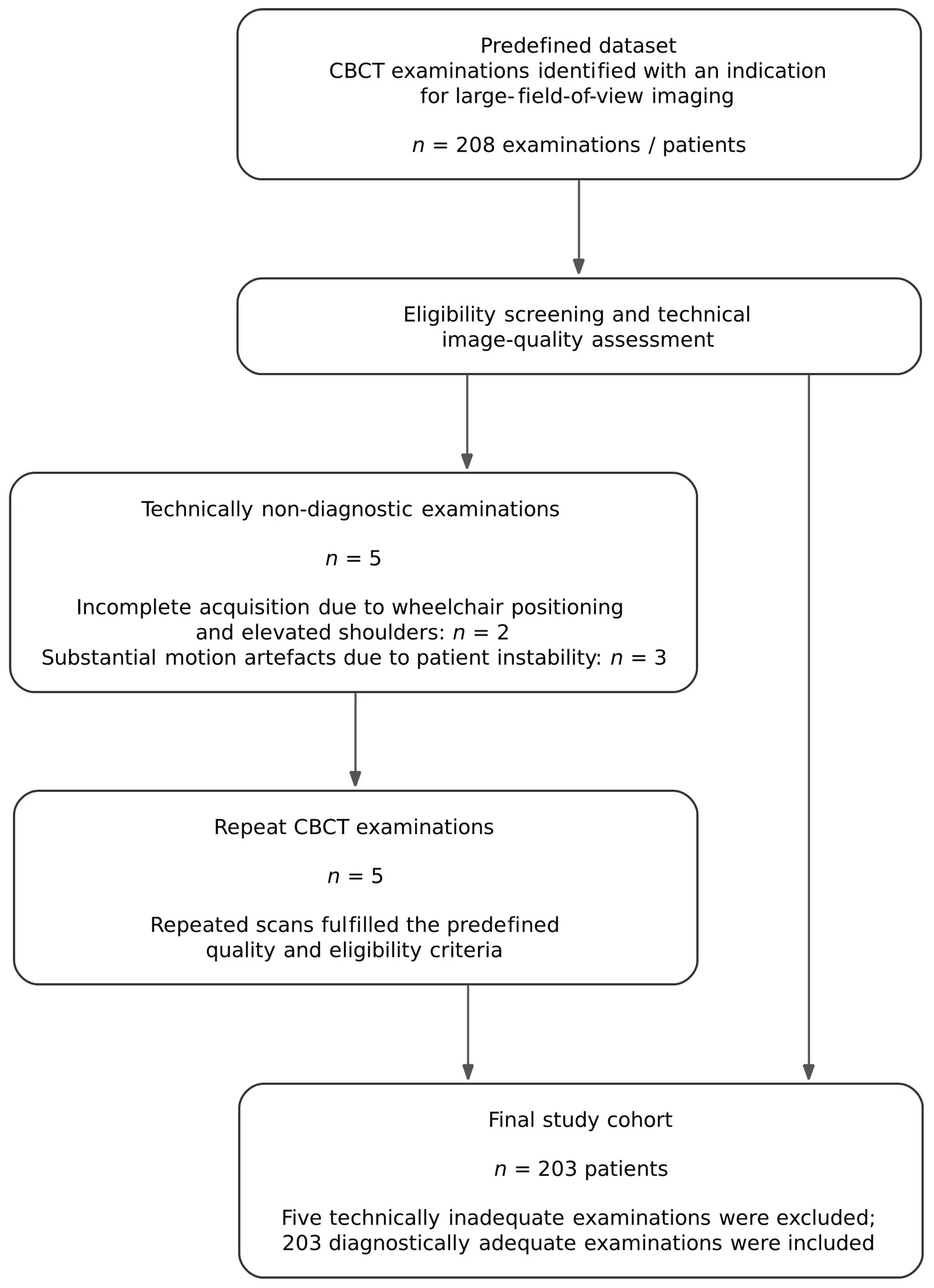
Flow diagram of study selection.

**Figure 2:**
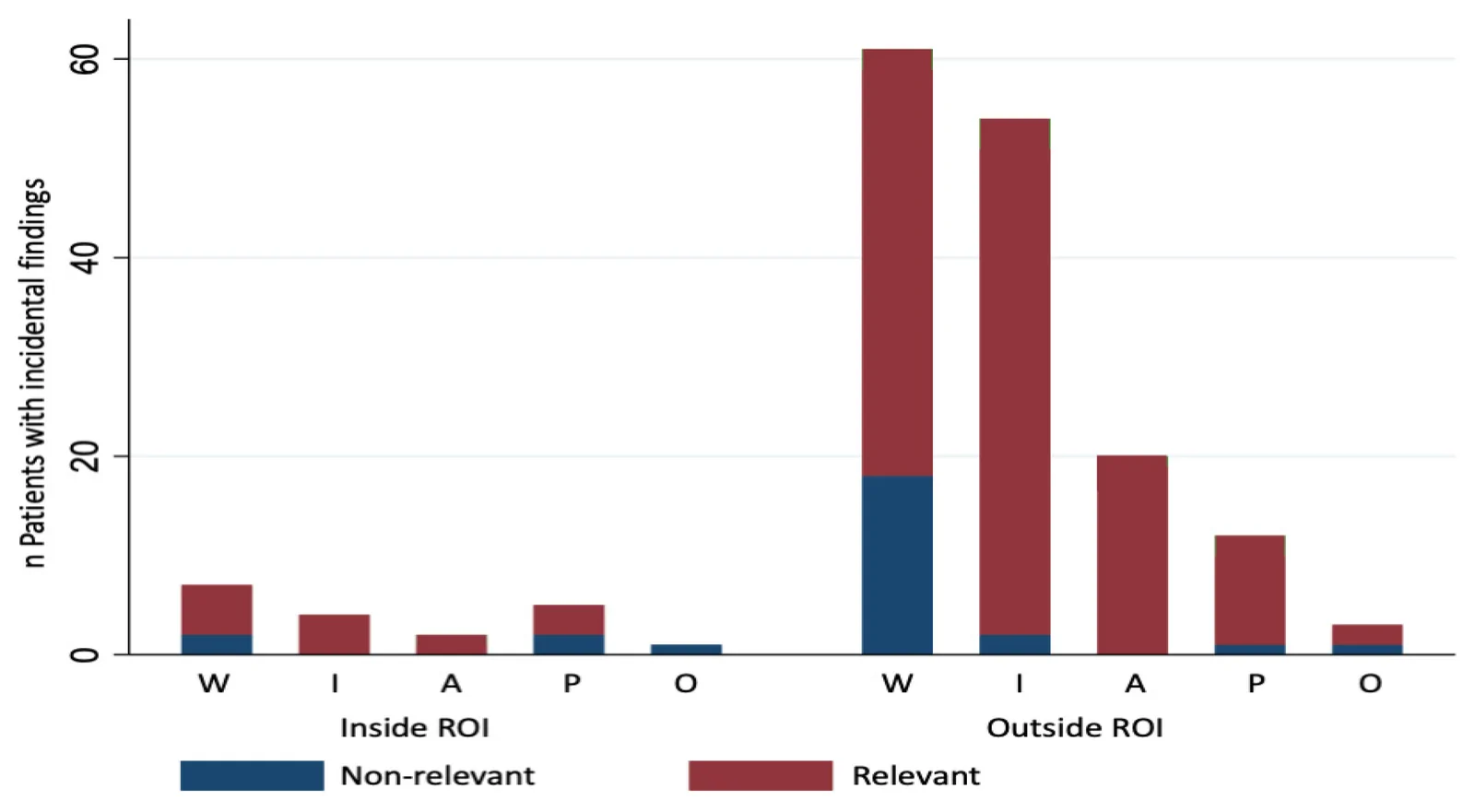
**Distribution of patients with incidental findings inside vs. outside the ROI, grouped by CBCT indication.** The classification into relevant versus non-relevant findings was based on whether the respective incidental finding required therapeutic intervention, clinical follow-up, or further diagnostic evaluation. CBCT = cone beam computed tomography; ROI = region of interest; W = wisdom teeth assessment; I = implant and augmentation planning; A = apical diagnostic; P = periodontal diagnostic; and O = orthodontic assessment.

**Figure 3:**
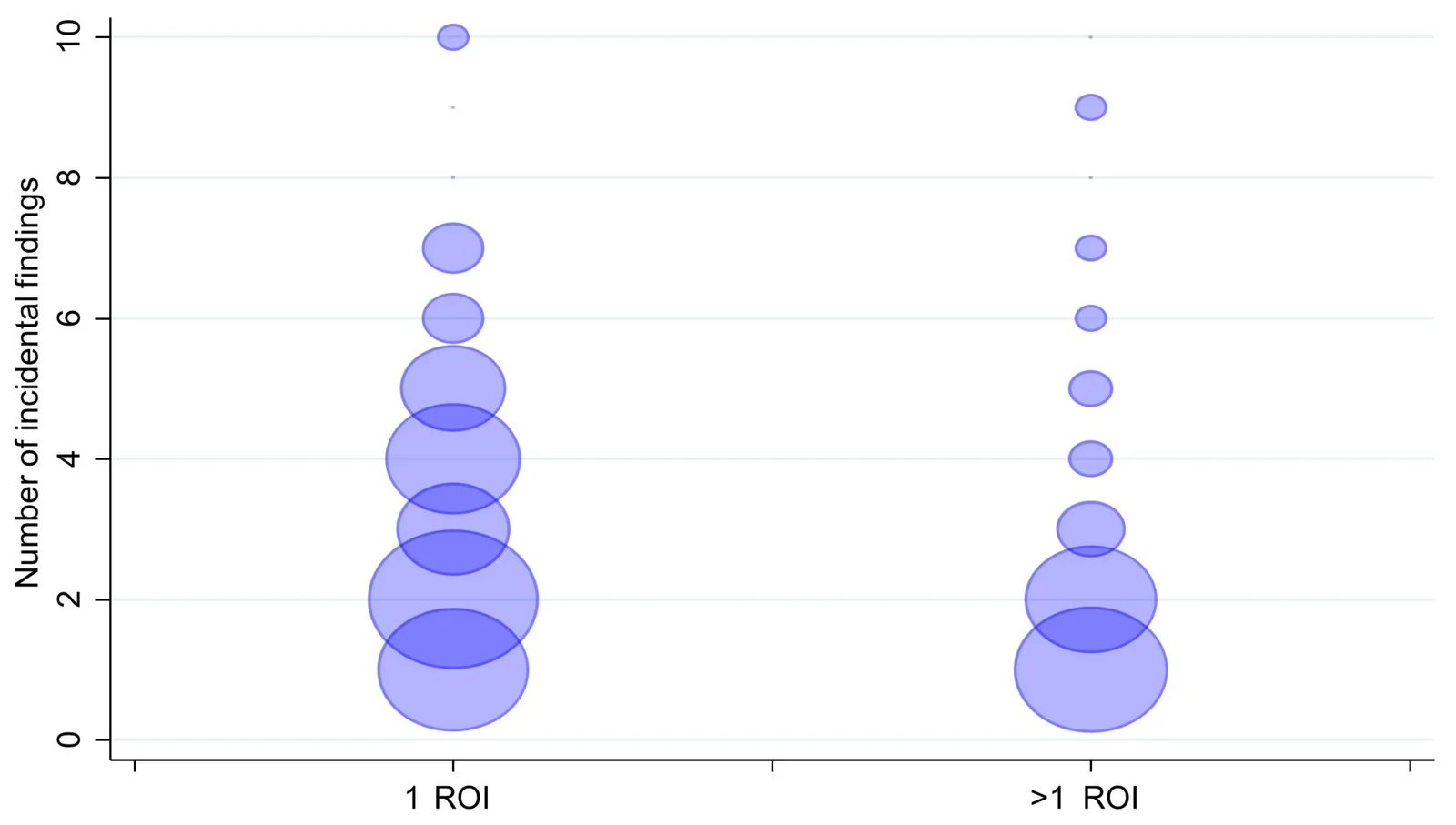
**Number of incidental findings for one or more ROIs under consideration.** The size of the circles is proportional to the number of findings.

**Figure 4:**
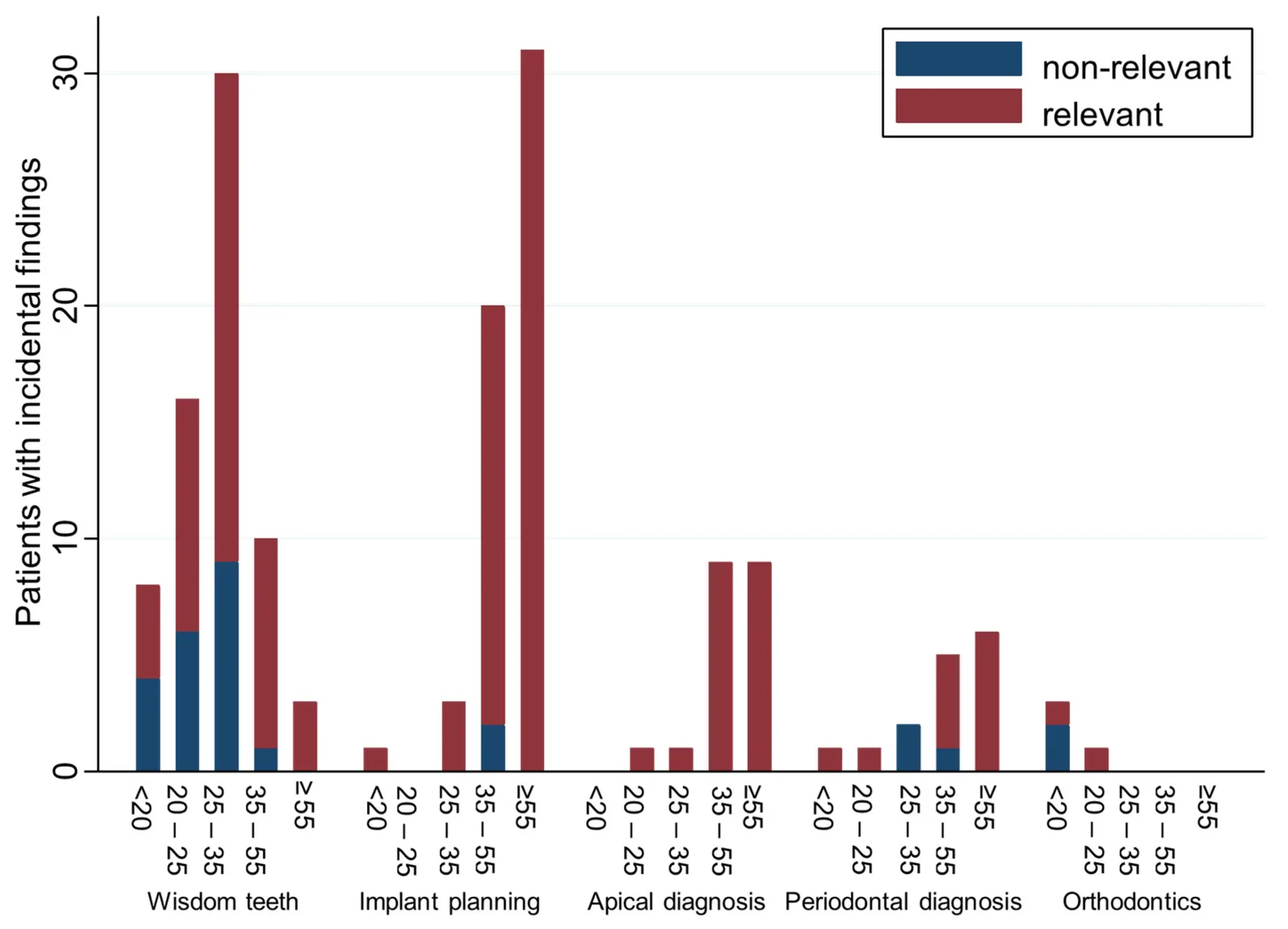
**Distribution of incidental findings in CBCT images by patient age classes, clinical relevance and CBCT indication.** The classification into relevant versus non-relevant findings was based on whether the respective incidental finding required therapeutic intervention, clinical follow-up, or further diagnostic evaluation. CBCT = cone beam computed tomography.

**Table 1:** Study population.

Characteristic	Total (*n* = 203)	Male (*n* = 80)	Female (*n* = 123)
Median age (years)	37	39	33
Mean age ± SD (years)	39.7 ± 17.7	42.0 ± 17.1	38.2 ± 18.1
Age range	18–79	18–79	18–78
CBCT indication			
Wisdom teeth	96	39	57
Implant planning	58	24	34
Apical diagnosis	24	10	14
Periodontal diagnosis	18	6	12
Orthodontics	7	1	6
Dental status			
Fully dentate	103	44	59
Partial dentition >24 teeth	58	19	39
Partial dentition ≤24 teeth	42	17	25

SD = standard deviation; CBCT = cone beam computed tomography.

**Table 2:** Incidental findings by category.

Finding Category	Patients with Findings	Percentage
Dental findings	106	52.2
Skeletal findings	44	21.7
Sinusoidal/airway findings	88	43.3
Other findings	80	39.4
Relevant findings (requiring treatment/follow-up/diagnosis)	134	66.0

Percentage refers to the total number of included individuals (*n* = 203).

**Table 3:** Incidental findings outside ROI.

Finding Category	Patients with Findings Outside ROI	Percentage of Findings Outside ROI
Dental findings	94	88.7
Skeletal findings	40	90.9
Sinusoidal/airway findings	82	93.2
Other findings	70	87.5
Relevant findings (requiring treatment/follow-up/diagnosis)	120	89.6

ROI = region of interest. Percentage refers to the total number of patients exhibiting incidental findings in the respective category.

**Table 4:** Prevalence of incidental findings by indication.

Indication	Patients with Incidental Findings	Percentage of Patients with Incidental Findings
Wisdom teeth	67	69.8
Implant planning	55	94.8
Apical diagnosis	20	83.3
Periodontal diagnosis	15	83.3
Orthodontics	4	57.1
Total	161	79.3

Percentage refers to the total number of patients within the respective indication group.

**Table 5:** Prevalence of incidental findings outside the ROI by indication.

Indication	Patients with Incidental Findings Outside ROI	Percentage of Patients with Findings Outside ROI
Wisdom teeth	61	63.5
Implant planning	54	93.1
Apical diagnosis	20	83.3
Periodontal diagnosis	12	66.6
Orthodontics	3	42.9

ROI = region of interest. Percentage refers to the total number of patients within the respective indication group.

**Table 6:** Distribution of incidental findings inside vs. outside the ROI by indication.

Indication	Patients with Incidental Findings Outside ROI	Patients with Incidental Findings Inside ROI	Percentage of Patients with Incidental Findings Outside ROI	*p*-Value
Wisdom teeth	61	6	91.0	<0.0001
Implant planning	54	1	98.2	<0.0001
Apical diagnosis	20	0	100	<0.0001
Periodontal diagnosis	12	3	80.0	<0.0001
Orthodontics	3	1	75.0	0.625

ROI = region of interest. Percentage refers to the total number of patients with incidental findings within the respective indication group.

**Table 7:** Association of demographic variables with relevant incidental findings.

Variable	Odds Ratio	95% Confidence Interval	*p*-Value
Age	1.11	1.06–1.17	<0.001
Gender (male vs. female)	0.93	0.37–2.30	0.874

**Table 8:** Pairwise comparisons of the number of incidental findings between CBCT indications based on the negative-binomial regression model.

Variable	IRR	95% Confidence Interval	Scheffé-Adjusted *p*-Value
Implant planning vs. third-molar assessment	2.81	1.98–3.97	<0.001
Apical assessment vs. third-molar assessment	1.98	1.23–3.20	0.001
Bone-related indication vs. third-molar assessment	1.96	1.15–3.34	0.004
Orthodontic assessment vs. third-molar assessment	0.77	0.23–2.54	0.977
Apical assessment vs. implant planning	0.71	0.45–1.10	0.216
Bone-related indication vs. implant planning	0.70	0.42–1.15	0.304
Orthodontic assessment vs. implant planning	0.27	0.08–0.89	0.023
Bone-related indication vs. apical assessment	0.99	0.54–1.80	1.000
Orthodontic assessment vs. apical assessment	0.39	0.11–1.32	0.226
Orthodontic assessment vs. bone-related indication	0.39	0.11–1.37	0.253

**Table 9:** Association of clinical variables with relevant incidental findings (univariate analysis).

Variable	IRR/OR	95% Confidence Interval	*p*-Value
Partial dentition >24 teeth vs. full dentition	IRR = 2.40	1.79–3.22	<0.001
Partial dentition ≤24 teeth vs. full dentition	IRR= 3.32	2.48–4.44	<0.001
≤24 teeth vs. >24 teeth	IRR = 1.38	1.05–1.81	0.014
Apicoectomized teeth	Not estimated	N/A	N/A
Root canal treated teeth	OR = 9.24	3.02–28.27	0.069
Non-periodontal vs. periodontal findings	OR = 2.17	0.45–10.54	0.336

N/A = not applicable.

## Data Availability

The data are not publicly available due to privacy and ethical restrictions related to participant confidentiality. The data presented in this study are available from the corresponding author upon reasonable request.

## Abbreviations

The following abbreviations are used in this manuscript:

ALADA	as low as diagnostically acceptable
ALARA	as low as reasonably achievable
CBCT	cone beam computed tomography
CI	confidence interval
FoV	field of view
GDPR	General Data Protection Regulation
IRR	incidence rate ratio
OR	odds ratio
ROI	region of interest
SD	standard deviation
